# Study on the Bioassay of Anti-Inflammatory Effects of Fuke Qianjin Capsule Based on COX-2 Inhibiting Activity

**DOI:** 10.1155/2021/6620124

**Published:** 2021-04-08

**Authors:** Run-ran Ma, Xiao-juan Yang, Ying Huang, Shuai-shuai Chen, Xiao-he Xiao, Jia-bo Wang, Ming Niu

**Affiliations:** ^1^College of Pharmacy, Henan University of Chinese Medicine, Zhengzhou, Henan 450000, China; ^2^China Military Institute of Chinese Medicine, Fifth Medical Center of Chinese PLA General Hospital, Beijing 100039, China; ^3^Beijing Tsinghua Changgung Hospital, School of Clinical Medicine, Tsinghua University, Beijing 102218, China; ^4^School of Traditional Chinese Medicine, Capital Medical University, Beijing 100069, China; ^5^Department of Poisoning Treatment, Fifth Medical Center of Chinese PLA General Hospital, Beijing 100071, China

## Abstract

Fuke Qianjin Capsule (FKQJ) is a common TCM compound formula in the treatment of gynecological inflammation-related diseases. This study intends to explore and establish a bioassay method to further improve its quality control. The bioassay method for the determination of anti-inflammatory biopotency was established based on its inhibitory activity on recombinant human cyclooxygenase-2 (COX-2), an active target of FKQJ in the treatment of female pelvic inflammatory disease. We firstly established chemical fingerprint of 20 batches of FKQJ by ultra-high-performance liquid chromatography to identify the components and analyze the chemical similarities. The similarity within different batches of FKQJ was relatively high. The values of similarity of the 19 batches were between 0.973 and 0.995, while one batch's similarity value was 0.813. Celecoxib, a selective inhibitor of COX-2, was chosen as the positive control drug in COX-2 activity assay to establish an anti-inflammatory biopotency detection method based on parallel line test of qualitative response. The methodological investigation showed that the method possessed good repeatability and precision. Secondly, the anti-inflammatory biopotency of 20 batches of FKQJ for inhibiting COX-2 was determined. The results showed that the biopotency of different batches of FKQJ ranged from 676 U/*μ*g to 1310 U/*μ*g, with average value of 918 U/*μ*g and RSD of 16.7%. Based on multiple linear regression analysis, we found that three contents were highly correlated with the anti-inflammatory biopotency, while chlorogenic acid was validated of the strongest anti-inflammatory activity in vitro. Compared with chemical detection, bioassay can better reflect the quality fluctuation of different batches of products and correlate the known pharmacodynamic targets. The supplement of the bioassay method based on chemical evaluation is helpful to improve the quality control ability of Chinese patent medicine and ensure its clinical efficacy is stable and controllable.

## 1. Introduction

Fuke Qianjin Capsule (FKQJ) is a commonly used traditional Chinese medicine in gynecology. It is composed of eight traditional Chinese medicines (TCM), namely, Moghaniae Radix, Mahoniae Caulis, Andrographis Herba, *Zanthoxylum dissitum* Hemsl, Spatholobi Caulis, Angelicae Sinensis Radix, Codonopsis Radix, and Rosae Lavigatae Radix. FKQJ is considered with the effect of clearing heat, eliminating dampness, replenishing vital energy (qi), and eliminating blood stasis in TCM theory and has significant efficacy in treating gynecological inflammatory diseases including chronic pelvic inflammatory disease, endometritis, and chronic cervicitis, in recent clinical practice [1]. Previous pharmacological studies have shown that FKQJ has significant anti-inflammatory effects and can effectively inhibit croton oil-induced ear swelling in mice, carrageenan-induced paw swelling in rats, cotton ball granuloma in rats, and uterine inflammation in rats [2]. However, FKQJ originated from folk prescription, in which Rosae Lavigatae Radix, Moghaniae Radix, *Zanthoxylum dissitum* Hemsl all conform to local standards. Currently, the chemical content indexes in quality control standards are only andrographolide and dehydrated andrographolide, which have limited representation on the quality of the whole prescription. Therefore, it is urgent to explore quality control methods correlated with curative effect for improving quality standards of FKQJ.

Bioassay method has been considered of prominent advantages in the evaluation and control of TCM quality because of the characteristics of multicomponent and overall effects of TCM, which makes up for the limitation of evaluating the quality of TCM by individual index components. Bioassay of TCM quality has been gradually regarded as an important direction for the development of quality control of TCM since the promulgation of the 2010 Edition of Chinese Pharmacopoeia. In 2015, the United States Food and Drug Administration (FDA) introduced the concept of bioassessment into the *Botanical Drug Development Guidance for Industry* [3]. A series of bioassay methods have been established for the quality of TCM, which are related to the biological activities of purgative, bacteriostasis, cardiotonic, hemostasis, antiviral, anti-inflammatory, antipyretic, anticoagulant effect, and so on, as well as minimum lethal toxicity, hepatotoxicity, and other biological toxicity evaluation methods [4], and have been successfully applied to TCM like Rhei Radix et Rhizoma [5], Lonicerae Japonicae Flos and Lonicerae Flos [6], Aconiti Lateralis Radix Praeparata [7], and Salviae Miltiorrhizae Radix et Rhizoma [8].

The analgesic, anti-inflammatory, and promoting blood circulation effects of FKQJ were reported to be closely related to cyclooxygenase-2 (COX-2) according to its mechanism in treating gynecological inflammatory diseases [9, 10]. COX-2 is a kind of major metabolic enzyme involved in arachidonic acid metabolism. Its downstream metabolites, prostaglandins and thromboxanes, can induce inflammation, fever, pain, blood coagulation, and other pathophysiological manifestations. COX-2 is one of the important targets of gynecological inflammatory diseases. Previous studies showed that FKQJ and several componential herbal medicines could inhibit the activity of COX-2 [11–13]. Therefore, we intend to explore and establish a bioassay method for the quality of FKQJ based on COX-2 inhibitory activity and compare them with the chemical analysis method, to comprehensively evaluate the quality fluctuation of different batches of FKQJ, which can provide insights for improving the quality control levels of FKQJ and other Chinese patent medicines.

## 2. Materials

### 2.1. Experimental Apparatus

Acquity ultra-performance liquid chromatography (Waters, USA); Synergy Mili-Q ultra-pure water preparation system (Millipore USA); desktop high-speed centrifuge (Thermo Fisher Scientific, USA); SynergyH2 full-function microplate detector (BioTek Instruments, USA) were used.

### 2.2. Medicine and Reagents

Fuke Qianjin Capsule was provided by Zhuzhou Qianjin Pharmaceutical Co. Ltd. The batch number information is shown in Table 1. The reference materials of 9 index components in FKQJ were provided by Chengdu Pufei De Biotech Co. Ltd.: chlorogenic acid (lot. 17101904, 99.76%); ferulic acid (lot. 17092501, 99.86%); genistin (lot. 17040702, 98.87%); jatrorrhizine hydrochloride (lot. 17031005, 98.92%); andrographolide (lot. 18011103, 99.62%); dehydrated andrographolide (lot. 170926, 98.25%); palmatine (lot. 18022602, 99.43%); berberine (lot. 17041901, 99.47%); ligustilide (lot. 18010201, 99.49%). Cyclooxygenase 2 Inhibitor Screening kit (supervised by China Military Institute of Chinese Medicine, produced by Shanghai Beyotime Co. Ltd., lot. 121819200522); DMSO (AMRESCO Inc., USA, batch number: 3304C252); Chromatographic methanol, acetonitrile, and phosphoric acid (Thermo Fisher Scientific, USA) were used.

## 3. Methods

### 3.1. Establishing the Chemical Fingerprint

#### 3.1.1. Chromatographic Conditions

Ultra-high-performance liquid chromatography (UPLC) (Waters, USA) was performed on A Waters ACQUITY HSS T3 column (2.1 mm × 100 mm, 1.8 *μ*m). The mobile phase was acetonitrile (solvent A) + 0.1% (v/v) phosphoric acid aqueous (solution B) with gradient elution as follows: 0∼4 min: 16% A; 4∼10 min: 16%∼19% A; 10∼20 min: 19%∼23% A; 20∼23.2 min: 23%∼31% A; 23.2∼31.2 min: 31%∼55% A; 31.2∼36.8 min: 55% A. The injection volume was 1.6 *μ*L, the flow rate was 0.20 mL/min, the detection wavelength was 254 nm, the column temperature was 30°C, and the column equilibrium time was 10 min.

#### 3.1.2. Preparation of Reference Solution

Chlorogenic acid, ferulic acid, genistin, jatrorrhizine hydrochloride, andrographolide, dehydrated andrographolide, palmatine, berberine, and ligustilide control substance were accurately weighed and added with methanol to prepare 1 mg/mL reserve solution, which was stored at 4°C for use.

#### 3.1.3. Preparation of Test Solution

FKQJ content was ground into fine powder—about 1 g fine powder accurately weighed, and 20 ml 75% methanol aqueous solution was added and weighed accurately. It was treated with ultrasound (power: 250 W, frequency: 40 kHz) for 30 min and then cooled at room temperature after precision weighing. The weight loss was supplemented by adding 75% methanol-water solution. The mixture was shaken and then filtered through a 0.22 *μ*m membrane. The filtrate served as the test solution for chemical fingerprint analysis.

#### 3.1.4. Similarity Evaluation

Chromatographic data of different batches of FKQJ were imported into similarity evaluation software (2012 edition, National Pharmacopoeia Committee) to calculate the similarity between batches.

### 3.2. Evaluation Method of COX-2 Activity

#### 3.2.1. Preparation of the Sample

The FKQJ sample was precisely weighed before use, and DMSO was added for ultrasonic dissolution. The mother liquor of 1 mg/ml was prepared at a certain volume and centrifuged, and the supernatant was filtered through 0.22-*μ*m ultrafiltration membranes. Nine monomeric components, including chlorogenic acid, ferulic acid, genistin, jatrorrhizine hydrochloride, andrographolide, dehydrated andrographolide, palmatine, berberine, and ligustilide, were accurately weighed before use. Assay buffer or DMSO was added for ultrasonic dissolution, and 4 mg/mL mother liquor was prepared at a certain volume and centrifugated, and the supernatant was filtered through 0.22-*μ*m ultrafiltration membranes. Test solutions of different concentrations were prepared by diluting with the buffer solution to test the inhibitory activity of COX-2.

#### 3.2.2. Determination of COX-2 Inhibition Rate

Blank control wells, 100% enzyme activity control wells, positive drug (selective COX-2 inhibitor celecoxib) control wells, and sample wells were set up, and each sample was set up in two parallel wells. The fluorescence intensity was detected after 5 min of incubation at 37°C. The excitation and the emission wavelength were 560 nm and 590 nm, respectively. The specific operation was following the instructions of COX-2 inhibitor screening kit. The fluorescence intensity of FKQJ solution with different concentrations and 9 monomer samples were tested to investigate the inhibitory effect of drugs on COX-2 enzyme activity.

#### 3.2.3. Methodological Study of COX-2 Activity Evaluation

The inhibition rate and relative standard deviation (RSD) of six samples of celecoxib at the same concentration were measured to evaluate the repeatability of the COX-2 activity evaluation method. The inhibition rate and RSD of celecoxib solutions of the same concentration on COX-2 activity in 6 repeated tests were measured in different time ranges, and intraday precision (within 1 day) and interday precision (6 consecutive days) were evaluated, respectively. The reproducibility was evaluated by the inhibition rate and RSD of celecoxib solutions of the same concentration on COX-2 enzyme activity, measured by different laboratory personnel.

#### 3.2.4. Biopotency Calculation of FKQJ Inhibiting COX-2 Activity

With celecoxib as the control substance, the inhibitory effects of FKQJ on COX-2 enzyme activity were calculated according to the “parallel line test of qualitative reaction” [14]. The biopotency was determined by the initial potency unit; therefore, it is the embodiment of relative value. To make the potency of the FKQJ reference substance easy to calculate and express, the biopotency of the FKQJ reference substance was defined as 1000 U·*μ*g^−1^ at first, and then the potency of celecoxib was determined, where the unit of potency U was given practical significance. The parallel model of quantal response was used to calculate the biopotency. The potency was calculated after the coordinate transformation of the inhibition rate and the corresponding administration concentration. Suppose the potency of the reference substance S and the test substance *T* are PS and PT, respectively. Firstly, line *M*, which is between the dose-effect parallel lines of the reference substance *S* and the test substance *T*, paralleled to the horizontal axis was obtained. The formula is as follows: $M={\bar{X}}_{s}-{\bar{X}}_{T}-\left({\bar{Y}}_{s}-{\bar{Y}}_{T}/b\right)$; therefore the potency of the test *substance* PT = Exp (*M*)·PS (*X*: logarithmic dose, *Y*: expect probit, *y*: working probit, *nw*: the weight of each reaction point) [15].

### 3.3. Correlation Analysis of Chemical and Bioassay Results

Pearson correlation and *multiple* linear regression analyses were used to investigate the correlation between the peak areas of 9 identifiable components and biopotency among different batches of FKQJ samples.

## 4. Results

### 4.1. Establishment of UPLC Fingerprint and Identification of Characteristic Peaks

**Figure 1:**
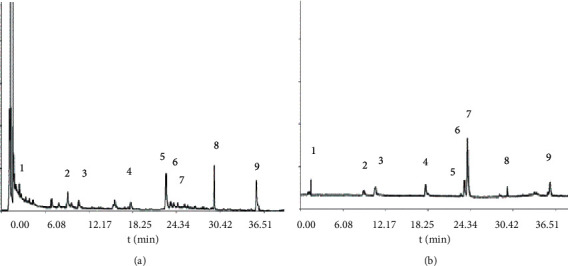
UPLC-UV chromatogram. (a) Sample. (b) Mixed control. 1: chlorogenic acid; 2: ferulic acid; 3: genistin; 4: jatrorrhizine hydrochloride; 5: andrographolide; 6: palmatine; 7: berberine; 8: dehydrated andrographolide; 9: ligustilide.

The typical chromatogram of FKQJ is shown in Figure 1. Nine components including chlorogenic acid, ferulic acid, genistin, jatrorrhizine hydrochloride, andrographolide, dehydrated andrographolide, palmatine, berberine, and ligustilide were determined by comparing the chromatograms of the test sample and the reference substances.

### 4.2. Fluctuation of the 9 Components in Different Batches of FKQJ

The peak areas of the 9 active components in 20 batches of FKQJ were determined. The results are shown in Table 1. The results indicate that different batches of FKQJ had marked variations in the content of each component. The variation coefficients of chlorogenic acid, ferulic acid, genistin, jatrorrhizine hydrochloride, andrographolide, palmatine, berberine, dehydrated andrographolide, and ligustilide were 11.34%, 19.14%, 20.19%, 30.79%, 18.19%, 16.43%, 36.88%, 29.02%, and 16.84%, respectively.

### 4.3. Similarity Analysis of Chemical Fingerprints

**Figure 2:**
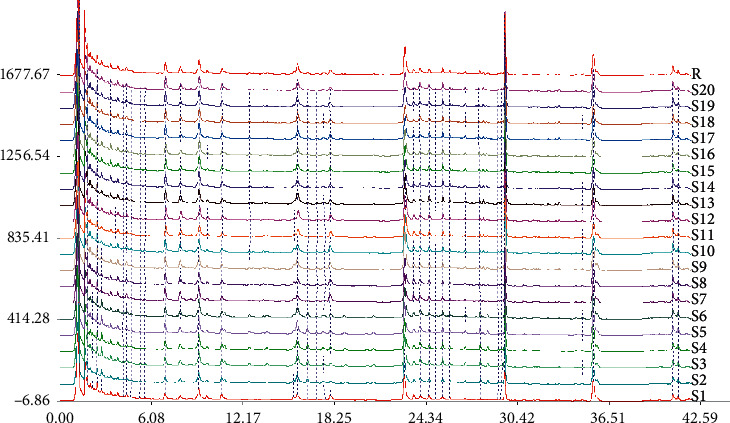
UPLC fingerprint of FKQJ.

The fingerprints (Figure 2) and similarity (Table 2) of 20 batches of FKQJ showed that the similarity of batch S5 was 0.813, and the similarity of the other 19 batches was between 0.973 and 0.995, indicating that the quality of FKQJ of each batch was stable under the evaluation of chemical analysis method.

### 4.4. Inhibition of COX-2 Activity by FKQJ

**Figure 3:**
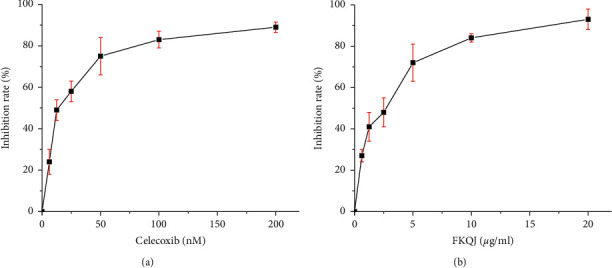
Inhibitory effects of celecoxib and FKQJ on COX-2 enzyme activity. (a) Representing the relationship between celecoxib and inhibition rate. (b) Representing the relationship between FKQJ and inhibition rate.

The fluorescence intensity of the treatment group was significantly lower than that of the 100% enzyme activity group (*P* < 0.01), indicating that FKQJ has a significant inhibitory effect on COX-2 enzyme activity in a dose-dependent range of 1.25∼25 *μ*g/mL, and the type and trend of the dose-response curve were the same as those of positive drug celecoxib (Figure 3).

### 4.5. Methodological Study

#### 4.5.1. Repeatability

**Figure 4:**
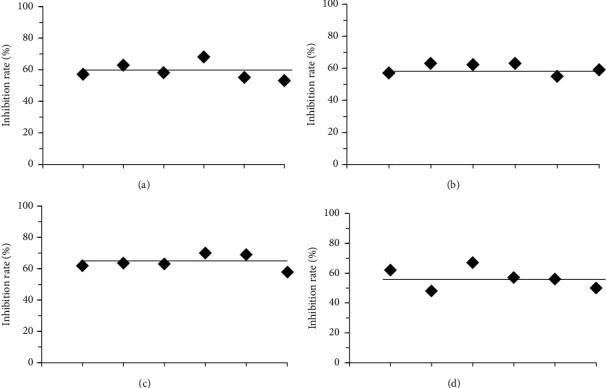
Methodological Investigation. The horizontal line in the figure shows the average inhibition rate of celecoxib against COX-2 activity. (a) Repeatability studies of inhibition rate of celecoxib on COX-2 activity. (b) Intraday precision studies of inhibition rate of celecoxib on COX-2 activity. (c) Diurnal precision studies of inhibition rate of celecoxib on COX-2 activity. (d) Reproducibility studies of inhibition rate of celecoxib on COX-2 activity.

Six samples were prepared from the same celecoxib solution with the same concentration (25 nM). Based on the above method, the average inhibition rate of celecoxib on COX-2 activity was 59.33% and RSD was 7.88%. The results are shown in Figure 4(a).

#### 4.5.2. Intraday Precision

The same celecoxib solution with the same concentration (25 nM) was taken, and its inhibitory effect on COX-2 was detected 6 times at different times on the same day according to the above method. The average inhibition rate of celecoxib on COX-2 activity was 60.23% and RSD was 5.13%. The results are shown in Figure 4(b).

#### 4.5.3. Interday Precision

The same celecoxib solution with the same concentration (25 nM) was taken and measured according to the above method. The inhibitory effects of celecoxib on COX-2 activity were detected for 6 days. The average inhibition rate of celecoxib on COX-2 activity was 64.50% and RSD was 6.56%. The results are shown in Figure 4(c).

#### 4.5.4. Reproducibility

The celecoxib solution of the same concentration (25 nM) from the same batch of kits was used to prepare the test solution, which was determined by different personnel from different laboratories following the above method. The average inhibition rate of celecoxib on COX-2 activity was 56.67% and RSD was 10.15%. The results are shown in Figure 4(d).

### 4.6. Biopotency Standardization of FKQJ Based on Positive Control Drug

After the coordinate conversion of inhibition rate and corresponding administration concentration, they were input into the potency calculation software to calculate the potency. Celecoxib was considered as the control drug, with the potency of celecoxib set at 1000 U/*μ*g. The potency of FKQJ was 2.145 U/*μ*g. The standardization data of FKQJ potency are shown in Table 3.

### 4.7. Biopotency of FKQJ in Inhibiting COX-2 Activity

**Figure 5:**
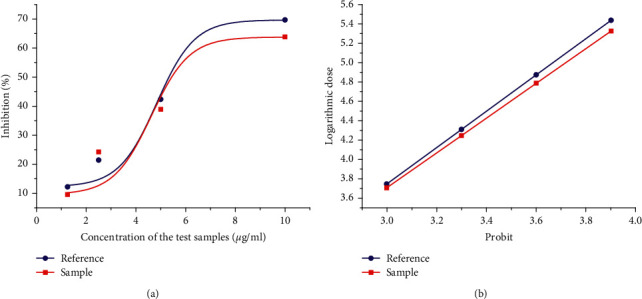
Coordinate transformation of dose and concentration. (a) Conversion of concentration and inhibition rate. (b) Conversion of logarithmic dose and probability.

**Figure 6:**
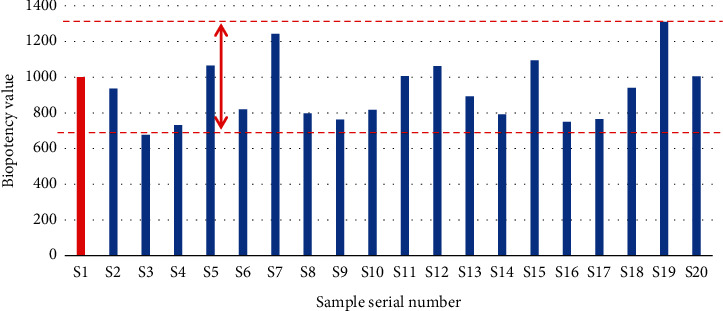
Biopotency value of FKQJ in different batches. The red dotted line represents the lowest and highest value of potency; the red column is the standard control batch.

Based on the above method, the inhibitory effects of FKQJ test product on COX-2 enzyme activity were detected, and the inhibition rate of each batch of FKQJ on the COX-2 enzyme activity was calculated. Considering S1 as the standard control, according to the conversion principle of mass reaction coordinate for biological verification, the inhibition rate and the corresponding dosing concentration coordinates were converted and then input into the potency calculation software to calculate the potency of each batch of FKQJ (Figure 5). The results showed that the potency of different batches of FKQJ ranged from 676 U/*μ*g to 1310 U/*μ*g, the highest potency was about twice the lowest potency, the average biopotency was 918 U/*μ*g, and the RSD was 16.7% (Figure 6).

### 4.8. Correlation Analysis of Bioassay and Chemical Evaluation

**Figure 7:**
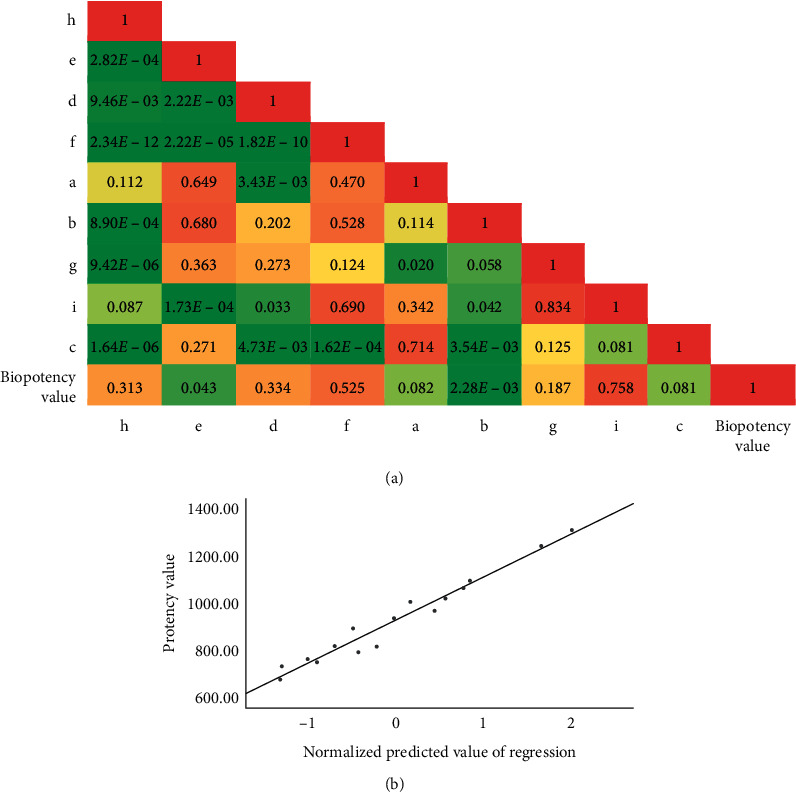
Correlation analysis between biological activity and chemical characteristic peaks of FKQJ. (a) Chlorogenic acid; (b) ferulic acid; (c) genistin; (d) jatrorrhizine hydrochloride; (e) andrographolide; (f) palmatine; (g) berberine; (h) dehydrated andrographolide; (i) ligustilide.

Univariate correlation analysis was performed on the relative potency in inhibiting COX-2 activity and 9 characteristic areas of FKQJ (Figure 7). Results indicate that, among the nine chemical indexes, ligustilide and palmatine highly correlate with anti-inflammatory biopotency in inhibiting COX-2 activity. It is suggested that the higher the content of ligustilide and palmatine, the higher the biopotency. Ligustilide and palmatine may be of the effective components of FKQJ to play an anti-inflammatory role. Furthermore, a multiple linear regression model (*P* = 0.002, *R*^2^ = 0.953) between 9 chemical components and biopotency was constructed, which indicated that 9 chemical components (*a*∼*i*) were significantly correlated with biopotency (*Y*). The model equation was *Y* = 2146.297 –0.893*a* + 0.831*b* − 1.334*c* + 0.724*d* − 1.028*e* + 2.852*f* + 0.256 *g*  + 0.098*h* − 0.278*i*. According to the standardized coefficient of a regression equation, palmatine (f), genistin (c), and andrographolide (e) have the highest correlation with biopotency. Among them, palmatine showed a high correlation in both univariate and multivariate correlation analysis, suggesting that it is an important active component in FKQJ that inhibits COX-2. Although the correlation coefficient was not high in univariate correlation analysis, genistin and andrographolide had a high correlation in multivariate correlation analysis, which suggested that these two components could be responsible for synergistic effects of FKQJ in inhibiting COX-2 activities. In addition, the normalization coefficients of *a*, *c*, *e*, and *i* were negative, suggesting that the inhibition of COX-2 activity may not be related.

### 4.9. Evaluation of the Anti-Inflammatory Activity of 9 Components in FKQJ

According to the COX2 kit method, the inhibitory effects of 9 components in FKQJ on COX-2 enzyme activity were tested. The coordinate transformation between inhibition rate and corresponding concentrations was input in the software to calculate the potency. Celecoxib was considered as the control drug, with a potency of celecoxib set at 1000 U/*μ*g; the monomer composition titer standardized data are shown in Table 4. The results showed that chlorogenic acid had the strongest anti-inflammatory activity, while andrographolide and dehydrated andrographolide only showed weak anti-inflammatory activity or no activity.

## 5. Discussion and Conclusion

By benefitting from the development of chromatographic analysis instruments and technology, quality control of TCM has made significant progress in recent decade. Most of the commonly used TCM have established quality control methods and standards based on the determination of the contents of index components [16–20]. Due to the complexity of the chemical composition of TCM and the relative hysteresis of the research of pharmacodynamic material basis, lots of the selected index chemical components are neither effective nor active. These properties cause certain limitations in the evaluation and control of the overall quality of TCM. And there is a weak association between the quality control method of TCM and their clinical efficacy and safety. It is also difficult to meet the growing demand for safe, efficient, and high-quality TCM products. TCM compound formula generally composes of a compound medicine containing numerous ingredients, which leads to difficulties in quality control. Therefore, it is necessary to select an appropriate quality control index according to clinical efficacy for the quality control of TCM compound formula.

In this paper, taking FKQJ as an example, based on its inhibitory activity on COX-2, which is an important target of gynecological inflammatory diseases, an anti-inflammatory biopotency detection method was established. The investigation of the dose-response relationship showed that the inhibitory effects of FKQJ on COX-2 enzyme activity were dose-dependent at the concentration of 1.25∼25 *μ*g/mL; the type and trend of the dose-response curve were the same as those of the positive drug celecoxib, a selective inhibitor of COX-2. The methodological investigation showed that the repeatability, intraday precision, interday precision, and reproducibility of the bioassay method of FKQJ for inhibiting COX-2 enzyme activity were reliable. The RSD of the bioassay method was less than 15%, which could meet the basic requirements for the bioassay of TCM quality. Furthermore, the anti-inflammatory potency of FKQJ was standardized by using the positive drug celecoxib as a reference. Moreover, the biopotency of 20 batches of FKQJ was determined by the parallel line test of qualitative reactions. Results showed that the potency of FKQJ ranged from 676 U/*μ*g to 1310 U/*μ*g, with an RSD of 16.7%. The results of similarity evaluation of chemical fingerprints showed that the similarity of 20 batches of products was high. Except for the similarity of one batch which was 0.813, the similarity of the other 19 batches was between 0.973 and 0.995. Taken together, the bioassay method can better reflect the overall quality fluctuation of different batches, compared to the chemical determination method. Based on the results of biopotency assay, we can trace back the reasons that affect the quality fluctuation of finished products of TCM drugs (such as the sources of raw materials, harvest time, processing methods, and production process parameters, etc.) and guide enterprises to improve the quality control in manufacture progress and thus improve the batch consistency of TCM drugs and to ensure the stability of clinical efficacy.

In this paper, the activity of the possible anti-inflammatory ingredients of FKQJ was also tested. The results showed that chlorogenic acid was the strongest active component in inhibiting COX-2 activity, and the other six components except for andrographolide and dehydrated andrographolide all have inhibitory effects on COX-2 activity in vitro. At present, andrographolide and dehydrated andrographolide regarded as quality control indexes of FKQJ in Chinese Pharmacopoeia are not directly related to anti-inflammatory activity, indicating that the current quality standards have limited ability to reflect the efficacy of finished products at least in the inhibition of COX-2 activity. FKQJ is originated from folk prescription, several herbal medicines of which still adopt the local standards. The research on pharmacodynamic substances and quality control standards needs to be strengthened. The biopotency detection method based on the COX-2 inhibitory activity proposed in this paper can guide the screening of active anti-inflammatory ingredients in FKQJ and its herbal medicines, thereby better clarifying its pharmacodynamic material basis. In addition, this method provides references for continuous improvement in quality control of the drug. In addition, FKQJ is usually prepared by boiling the eight kinds of TCM in water, and it may not be the most effective method for extracting active components from the TCM. The emerging potential methods of pretreatment and extraction of active components with hydrogen peroxide presoaking may bring unexpected insights into the material basis of FKQJ [21].

## Figures and Tables

**Table 1:** Peak area of nine components in FKQJ.

Sample		Peak area								
Number	Batch number	Chlorogenic acid	Ferulic acid	Genistin	Jatrorrhizine hydrochloride	Andrographolide	Palmatine	Berberine	Dehydrated andrographolide	Ligustilide
S1	20170226	984.6	839.5	423.7	336.3	1 473.5	355.7	428.4	930.3	1 121.0
S2	20170406	951.4	657.5	207.7	170.6	1 528.6	293.6	512.6	1 175.4	995.5
S3	20170408	1 372.9	1 149.6	336.0	252.9	2 049.1	383.6	798.3	1 726.2	1 019.6
S4	20170410	1 419.6	1 440.9	461.3	344.9	2 018.1	396.0	779.1	1 481.1	1 503.6
S5	20170411	1 074.6	1 472.5	466.4	338.7	2 016.2	410.2	800.7	1 489.1	1 481.7
S6	20170412	1 335.9	1 134.7	263.4	272.6	2 011.0	416.2	794.2	1 473.0	1 467.0
S7	20170518	1 178.9	1 076.4	453.0	525.3	1 835.8	499.2	626.6	1 261.0	1 271.0
S8	20170520	1 126.1	979.4	438.3	514.5	1 789.7	485.9	601.0	1 216.2	1 297.7
S9	20170523	1 151.8	1 036.9	327.2	489.2	2 436.6	476.5	585.0	2 462.4	1 429.5
S10	20170526	1 139.4	1 031.0	302.7	533.0	2 271.4	422.2	457.5	2 146.0	990.0
S11	20170530	993.9	740.6	293.3	421.4	1 809.3	346.0	378.1	1 890.5	855.7
S12	20170535	1 236.3	1 019.9	371.2	397.4	2 001.8	358.2	417.8	2 292.0	1 380.1
S13	20170603	1 244.2	1 088.1	274.3	314.6	1 870.4	311.7	320.8	2 962.7	1 080.5
S14	20170620	1 257.9	1 188.3	315.9	274.1	1 799.6	351.5	312.6	2 024.7	1 321.2
S15	20170621	938.2	933.9	342.5	278.1	1 690.4	358.1	314.2	2 128.2	1 145.0
S16	20170622	1 155.5	929.4	319.1	256.0	1 678.1	328.3	293.3	1 891.0	1 272.1
S17	20170624	1 184.4	1 156.2	390.7	303.2	1 493.5	391.0	348.0	2 413.7	1 669.0
S18	20170626	1 236.2	1 034.6	377.1	249.0	1 265.0	300.6	685.4	2 520.8	1 196.1
S19	20170627	1 196.6	967.5	317.2	332.4	1 205.5	328.6	292.9	2 385.3	1 478.2
S20	20170703	1 200.5	861.5	343.0	238.4	1 369.1	300.1	781.9	2 531.1	1 411.2
C.V.%		11.3	19.1	20.2	30.8	18.2	16.4	36.9	29.0	16.8

**Table 2:** Similarity analysis of UPLC fingerprint.

Number	Similarity
S1	1
S2	0.988
S3	0.991
S4	0.983
S5	0.813
S6	0.984
S7	0.995
S8	0.994
S9	0.986
S10	0.983
S11	0.981
S12	0.984
S13	0.973
S14	0.985
S15	0.98
S16	0.985
S17	0.979
S18	0.977
S19	0.976
S20	0.977

**Table 3:** Potency conversion of FKQJ and celecoxib.

No.	FKQJ		Celecoxib	
	Dose (*μ*g·mL^−1^)	Inhibition rate (%)	Dose (ng·mL^−1^)	Inhibition rate (%)
1	1.25	12.42	2.38	13.54
2	2.50	25.76	4.77	24.09
3	5.00	56.76	19.07	74.33
4	10.00	72.39	76.28	91.50

**Table 4:** Potency conversion between 9 monomer components and celecoxib.

Component	Potency value	FL%	*R*
chlorogenic acid	3.182	3.93	0.381
Genistin	1.158	1.46	0.116
Ferulic acid	0.759	0.68	0.076
Jatrorrhizine hydrochloride	0.095	0.09	0.010
Berberine	0.077	0.08	0.008
Ligustilide	0.021	0.02	0.002
Palmatine	0.008	0.01	0.001

## Data Availability

The data used to support the findings of this study are available from the corresponding author upon request.
